# Microtopography of Immune Cells in Osteoporosis and Bone Lesions by Endocrine Disruptors

**DOI:** 10.3389/fimmu.2020.01737

**Published:** 2020-09-02

**Authors:** Roberto Toni, Giusy Di Conza, Fulvio Barbaro, Nicoletta Zini, Elia Consolini, Davide Dallatana, Manuela Antoniel, Enrico Quarantini, Marco Quarantini, Sara Maioli, Celeste Angela Bruni, Lisa Elviri, Silvia Panseri, Simone Sprio, Monica Sandri, Anna Tampieri

**Affiliations:** ^1^Laboratory of Regenerative Morphology and Bioartificial Structures (Re.Mo.Bio.S.), Department of Medicine and Surgery - DIMEC, Unit of Biomedical, Biotechnological and Translational Sciences (S.BI.BI.T.), Museum and Historical Library of Biomedicine - BIOMED, University of Parma, Parma, Italy; ^2^OSTEONET-CMG Unit (Osteoporosis, Nutrition, Endocrinology, and Innovative Therapies) at the Medical Center Galliera (CMG), San Venanzio, Italy; ^3^Interdepartment Center for Law, Economics, and Medicine of Sport, University of Parma, Parma, Italy; ^4^Division of Endocrinology, Diabetes, and Metabolism, Department of Medicine, Tufts Medical Center, Tufts University School of Medicine, Boston, MA, United States; ^5^CNR- National Research Council of Italy, Institute of Molecular Genetics “Luigi Luca Cavalli-Sforza” - Unit of Bologna, Bologna, Italy; ^6^IRCCS Istituto Ortopedico Rizzoli, Bologna, Italy; ^7^Food and Drug Department, University of Parma, Parma, Italy; ^8^ISTEC - CNR, Faenza, Italy

**Keywords:** osteoporosis, organoid, immunomodulation, bone remodeling, endocrine disrupting chemical, immunobiology

## Abstract

Osteoporosis stems from an unbalance between bone mineral resorption and deposition. Among the numerous cellular players responsible for this unbalance bone marrow (BM) monocytes/macrophages, mast cells, T and B lymphocytes, and dendritic cells play a key role in regulating osteoclasts, osteoblasts, and their progenitor cells through interactions occurring in the context of the different bone compartments (cancellous and cortical). Therefore, the microtopography of immune cells inside trabecular and compact bone is expected to play a relevant role in setting initial sites of osteoporotic lesion. Indeed, in physiological conditions, each immune cell type preferentially occupies either endosteal, subendosteal, central, and/or perisinusoidal regions of the BM. However, in the presence of an activation, immune cells recirculate throughout these different microanatomical areas giving rise to a specific distribution. As a result, the trabeculae of the cancellous bone and endosteal free edge of the diaphyseal case emerge as the primary anatomical targets of their osteoporotic action. Immune cells may also transit from the BM to the depth of the compact bone, thanks to the efferent venous capillaries coursing in the Haversian and Volkmann canals. Consistently, the innermost parts of the osteons and the periosteum are later involved by their immunomodulatory action, becoming another site of mineral reabsorption in the course of an osteoporotic insult. The novelty of our updating is to highlight the microtopography of bone immune cells in the cancellous and cortical compartments in relation to the most consistent data on their action in bone remodeling, to offer a mechanist perspective useful to dissect their role in the osteoporotic process, including bone damage derived from the immunomodulatory effects of endocrine disrupting chemicals.

## Introduction

**Figure 1:**
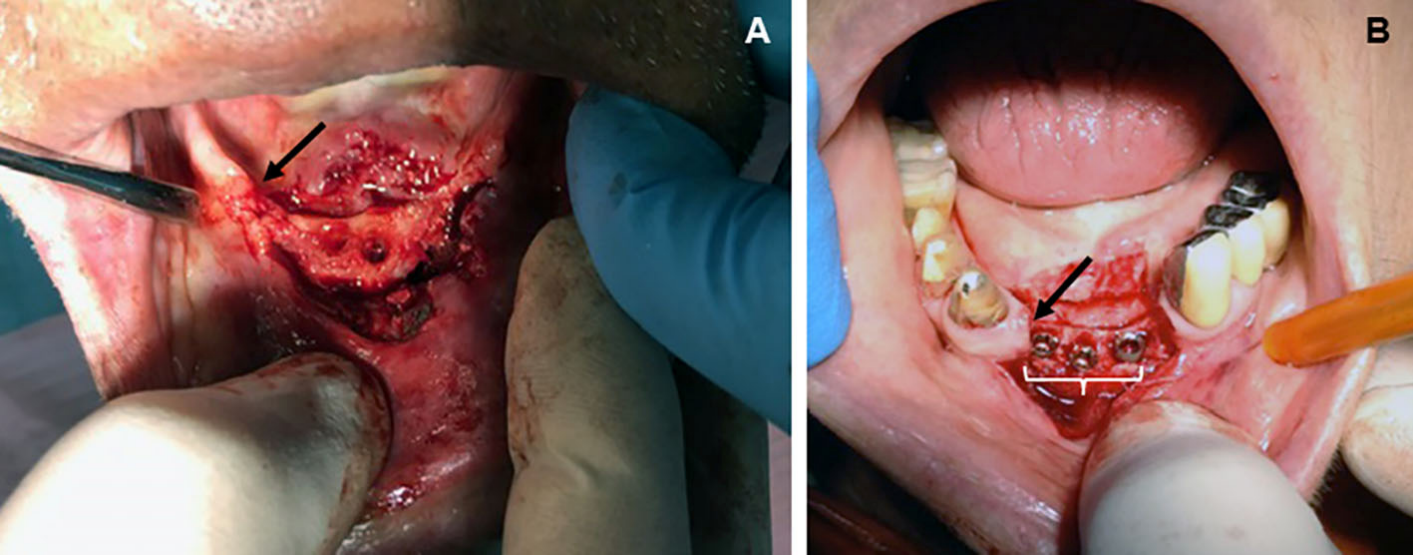
Topographic distribution of bone lesions in the human jaw following classical osteoporotic processes (chronic bacterial inflammation and menopause). **(A)** Edentulous patient (male, age 74 years) showing osteoporotic vertical resorption (arrow) of the maxilla during severe periodontitis. Surgical displacement of the gum flap revealed consistent porosity of the vast majority of the exposed bone, including complete loss of the cortical bone but more limited destruction of the cancellous trabeculae; **(B)** loss of central and lateral, inferior incisors in a patient (female, age 74 years) with vertical resorption (arrow) of the jaw as a consequence of severe postmenopausal osteoporosis. Note the presence of bone implants (white bracket) becoming visible after exposing the resorbed bone through a gum flap. Osteoporosis led to a decreased rate of cancellous bone formation in both the implanted socket and interdental bone, thus increasing risk of trabecular microfractures and prosthetic instability (from the Odontostomatological Archive of CMG, San Venanzio di Galliera, BO, Italy, with permission).

Osteoporosis is a worldwide public health problem, primarily as a result of increasing survival in aging (1), involves an estimated 200 million people worldwide (2), and has a global economic impact estimated up to more than 20 billion euros per year during the next 5 years on the health care systems of Western countries (3). By definition, osteoporosis is a systemic skeletal disease characterized by decreasing bone mass and microarchitectural deterioration of bone tissue that leads to an increased risk of bone fragility and fracture (4). Osteoporotic changes worsen in postmenopausal females and can variably affect any bone, but more frequently the femoral neck, vertebrae, and distal radius where unique patterns of bone derangement emerge. This lesional microtopography is now gaining particular interest in bones traditionally considered less affected by osteoporosis such as the jaw (Figures 1A,B) because of the increased request of prosthetic implants (5) and as a unique site for chronic inflammation and aseptic osteonecrosis during antiresorptive therapy (6).

Different cellular and molecular mechanisms may lead to osteoporosis: however, the low-grade systemic inflammation associated with aging is emerging as a critical stimulus for diffuse bone loss and reduced bone regenerative potential. At the same time, it highlights the role of the immune cells to favor resorption and reduce deposition of mineral mass, as well as to hamper the action of bone progenitors (7). Immune cells residing in the bone may contribute to development of both primary (postmenopausal, senile) and secondary (autoimmune, infective, vascular, neurological, endocrine, and multiorgan failure) osteoporosis acting on the progenitors of osteoblasts (OBs) and osteoclasts (OCs) (8). Finally, beyond pharmacological treatments well-known to interfere with OBs and OCs, endocrine-disrupting chemicals (EDs) may affect the activity of the immune cells in bone, leading to bone weakness (9).

To shed light on the interplay between immune and other bone cells in early osteoporotic changes to the cancellous and/or cortical bone, we here analyze the selective segregation of immune cells in different bone compartments in basal and activated states. We also discuss this microtopography in relation to the best established views on the effect of immune cells in bone remodeling, both in physiological conditions and upon an either inflammatory or toxic trigger. Collectively, we offer a mechanistic and space-related perspective of the action of immune cells involved in the osteoporotic process.

## Microtopography of Immune Cells in Bone Compartments

Osteoporotic lesions exhibit a well-defined pattern in the different bone compartments, the topography of which is time-dependent: (1) resorption of cancellous trabeculae in the epiphysis of long bones, vertebrae, other short and flat bones, cranial diploe; (2) subendosteal and, later, subperiosteal resorption of the cortical lamellae, primarily in long bones; (3) intracortical resorption with thinning of the entire compact tissue, primarily in long bones (10). It is therefore clear that peculiar microanatomical conditions selectively expose specific bone sites to the osteoporotic damage following a temporal progression. The microtopography of the immune cells in the bone compartments reveals that their original location and triggered recirculation are consistent with the microanatomical specificities of the osteoporotic damage as described above.

### Monocytes, Osteal Macrophages or Osteomacs, and Mast Cells

In the cancellous compartment of the epiphyses of long bones, and of flat and short bones, monocytes, and osteal macrophages or osteomacs (OMCs) are part of the mononuclear cells of the bone marrow (BM). Upon activation, monocytes migrate from the central to the perisinusoidal BM to enter the vascular sinuses and the venous capillaries *en route* to the cortical bone through the Haversian and Volkmann canals, up to the general circulation (11). This transfer pathway is supported by evidence that synthesis of interleukin 17 (IL-17) is increased both in BM cells and peripheral blood mononuclear cells during postmenopausal osteoporosis (12), suggesting a common BM source for both cell types.

In contrast, activated OMCs including proinflammatory M1 and anti-inflammatory M2 (13) may migrate from centers of erythropoiesis in the erythroblastic islands coincidental with the reticular niches of the central and perisinusoidal BM (14, 15) to the endosteal and subendosteal BM (i.e., tissue adjacent to main bulk of BM), to support myelopoiesis from hemopoietic stem cells (HSCs) and uncommitted progenitors (16). Therefore, an inflammatory trigger pushes monocytes and OMCs toward opposite directions inside the BM. Parts of the OMCs are present also in the connective periosteal layer of the compact bone (17). In this location, they are in a position to favor the shuttling of interstitial fluids from the extracellular matrix (ECM) of compact lamellae inside the periosteal lymphatics (18), thus preventing the detrimental mechanical effect of ECM fluid overloading on the cortical mineral mass (19).

Finally, mast cells are concentrated in the hematopoietic niche of the metaphyseal perisinusoidal BM, with some residing as flattened cells on the epiphyseal and diaphyseal endocortical surface. In osteoporotic bones, mast cells are found in close proximity to OCs, suggesting their massive migration to the endosteal BM (20).

### T and B Lymphocytes and Dendritic Cells

T and B lymphocytes variably account for up to 20% of BM mononuclear cells (21, 22). In the spongy bone, the majority of T lymphocytes are distributed: (A) throughout the reticular argyrophilic stroma, contributed by the ramified processes of the adventitial reticular cells (so-called CAR cells in the mouse) enwrapping the vascular sinusoids in the perisinusoidal BM (23), and (B) in the hemopoietic parenchyma, condensed in lymphoid follicle-like structures (24) in the central BM (25). However, 1/3 T cells are CD4^+^ CD25^+^ regulatory T (Treg) lymphocytes that reside in both the perisinusoidal and endosteal BM (26). When T cells get activated, they are generally found condensed in the endosteal and subendosteal BM (also known as HSC area) to interact with endothelial capillary cells and sinusoid-derived pericytes (16).

Differently, both differentiation and activation of B cells seem to be constrained within the reticular niches of the central and perisinusoidal BM, as suggested by their obligatory interaction with IL-7–secreting cells selectively diffused to the same BM compartments (16, 27).

Finally dendritic cells are spread inside all four BM compartments, in perivascular locations including arterioles and venous sinuses, and in close contact to both endothelial and adventitial reticular cells (28).

## Key Points on Immunobiology of Bone Remodeling Relevant to the Microtopography of Immune Cells in Bone Compartments

In physiological conditions, all aforementioned immune cells contribute to bone growth and mineralization. However, in the vast majority of osteoporotic forms (primary and secondary), the immune cells induce overactivation of OCs coupled with a reduction in OB activity.

### OMCs and Mast Cells

The OMCs mediate the transition between innate and adaptive immune responses. They have been found to be associated mainly with endosteal and, to a lesser extent, periosteal surfaces where they regulate maturation, function, and survival of OBs, collectively ensuring bone development, homeostasis, and repair (17, 29). Specifically, anti-inflammatory OMCs or M2 favor mineral deposition through formation of a canopy over mature matrix-producing OBs at sites of bone remodeling, and inhibit osteoclastogenesis through the action of IL-4 and IL-10 (30, 31). Differently, in a bone microenvironment characterized by chronic inflammation, proinflammatory OMCs or M1 stay close to endosteal OCs, and both cells respond to the lineage-specific growth factor macrophage colony-stimulating factor (CSF) released by mesenchymal stromal cells (MSCs) and OBs. As a result, OMCs secrete IL-1β, IL-6, and tumor necrosis factor (TNF) α catalizing differentiation of pre-OCs to functionally competent OCs, thus inducing bone resorption (30–32). A further level of OCs activation by M1 is provided through release of extracellular microvesicles containing histones (13). It is therefore expected that primary sites of lesion ensuing from action of OMCs are the trabecular subendosteal bone and internal free edge of the lamellae in the cortical case, only later involving the periosteal surface. Similarly, activated mast cells promote osteoclastogenesis by releasing histamine, TNF-α, and IL-6, and inhibit osteoblastogenesis primarily through secretion of IL-1 (20). Thus, it is expected that they initially induce resorption of the metaphyseal endosteum, only later involving the internal free edge of the cortical bone in both epiphysis and diaphysis.

### T Cells

T cells represent a major player in the adaptive responses of bone to pathogens, accounting for the majority of resident lymphocytes. Upon activation, T cells strongly promote pre-OCs differentiation by secreting TNF-α and RANKL; consequently, they are involved in many forms of osteoporosis (33, 34). This action is boosted by TNF-α, IL-1, IL-17, and IL-18 secreted by surrounding cells (OMCs, OBs), which lead to upregulation of RANKL expression by T cells (7, 35). Then, T cell–dependent secretion of interferon γ (IFN-γ) enhances formation of OCs and bone resorption, further favoring T cell–dependent cosecretion of TNF-α and RANKL under estrogen deficiency and infection (36). This explains the occurrence of osteoporosis in conjunction with chronic inflammatory disorders such as periodontitis in the postmenopausal female (37).

Among the various T-cell subpopulations, T helper 17 (T_H_17) lymphocytes secrete IL-17 able to induce RANKL expression by OBs and synovial fibroblast, and TNF-α and IL-1 by synovial macrophages, promoting OC formation (38). In addition, IL-17 secreted by peripheral blood mononuclear cells directly stimulates human osteoclastogenesis, favoring formation of actin rings in mature OCs (39). Finally, T_H_17 lymphocytes are downregulated by estrogens; thus, menopausal estrogen deficiency promotes local upregulation of T_H_17 (40). Consistently, the number of T_H_17 cells and levels of IL-17 in peripheral blood are increased in postmenopausal osteoporosis (12). Differentiation and expansion of T_H_17 cells are also favored by a number of resident cells including (1) OBs, OMCs, and stromal cells of the osteogenic layer of the periosteum and endosteum, through local secretion of IL-1 and IL-6; (2) OBs and OCs via release of transforming growth factor α and bone morphogenetic proteins, the latter partly available in the bone ECM as latent stored proteins [so-called “crinopexic” molecules, a term originally proposed by the Nobel Laureate Roger Guillemin, see (41)]; (3) BM dendritic cells and OMCs by means of IL-23 (42, 43).

In contrast to a resorptive action, T cells may also exert an inhibitory regulation of osteoclastogenesis through the CD137–CD137L complex. Indeed, CD137 is a costimulatory member of the TNF receptor family induced by T-cell receptor activation on T cells, whereas CD137L is its ligand expressed on BM dendritic cells and OCs precursors. *In vitro*, the CD137–CD137L complex suppresses OCs activation by inhibiting the multinucleation process (44). Similar, T cell–dependent release of IFN-γ may *in vitro* block RANKL signaling and suppress OC formation (36). Finally, the anti-inflammatory subpopulation of Treg cells (45) may inhibit OC differentiation via intercellular contacts mediated by cytotoxic T-lymphocyte-associated protein 4 and release of IL-4 and IL-10 (46). For this reason, their stimulation ameliorates osteolytic bone destruction (24). Collectively, mice lacking T cells have osteoporotic bones, suggesting a contribution of T lymphocytes in maintaining bone homeostasis during basal physiology (47).

In summary, upon T-cell activation, primary expected sites of lesion are the subendosteal cancellous bone and the internal free edge of the cortical bone. However, thanks to their spread through veins efferent from the BM to the Haversian and Volkmann canals, T cells can then reach the inner part of osteons; here, pathological cortical porosity occurs as a result of local resorption, collapse and fusion of adjacent Haversian canals, and disappearance of osteocyte lacunae (10, 48). Only later, activated T lymphocytes may also trigger reabsorption of subperiosteal compact bone.

### B Cells

B lymphocytes are a primary constituent of the hematopoietic niche of the BM (49), where recruitment and maturation of B-cell progenitors are controlled by adventitial reticular cells (positioned to enwrap the BM sinusoids) through release of CXCL12 (CXC chemokine ligand 12 or stromal cell–derived factor 1) (23, 47, 50). During an inflammatory state, B cells remaining attached to the central BM region may act as a source of RANKL to cause endosteal OC activation and bone resorption (51); equally, in postmenopausal osteoporotic patients, they release granulocyte-macrophage CSF to promote differentiation of OCs precursors (52).

In contrast, in physiological conditions the entire B lineage including multiple subsets of B-cell precursors, immature B cells, and plasma cells may act as inhibitory regulators of the RANK/RANKL system (53) by producing 64% of total BM osteoprotegerin (OPG). Thus, B cells basically favor synthesis of bone matrix and mineral deposition (47) that, conversely, are inhibited in the B cell–knockout mice resulting deficient in BM OPG and hence osteoporotic (51).

In conclusion, osteoporotic recruitment of B cells may lead to a double phase of catabolic and anabolic action in the bone: an initial resorption of the entire thickness of the cancellous lamellae followed by compensatory bone deposition, as reported to occur in the trabecular bone of the radius in postmenopausal osteoporosis (54).

### Dendritic Cells

Dendritic cells are professional antigen-presenting cells that in the BM are better known as type 2 conventional dendritic cells (cDC2). They regulate memory of local T cells, promote survival of recirculating mature B cells, and by interacting with endothelial and adventitial reticular cells mobilize HSC, uncommitted progenitors, and OMCs in the hemopoietic niche (28). From the point of view of a local information system, they look like a multiplexer able to select numerous inputs toward a single output (either a prevalent immune or osteoregulatory response). Indeed, similarly to activated T cells, they may release RANKL to elicit osteoclastogenesis and bone resorption in response to an inflammatory osteoporotic insult (7); in contrast, similarly to B lymphocytes, cDC2 may participate in the regulation of physiological bone remodeling by secreting the RANKL decoy receptor OPG (53) and thus inhibit activation of OCs. As a result, during an osteoporotic trigger, cDC2 may act as a “buffer system” to counterbalance the prevailing osteocatabolic effects of each immune cell type toward an osteoanabolic one.

## Role of EDs as Inducers of Osteoporosis Via Immunomodulation

Endocrine-disrupting chemicals are substances with an endocrine mode of action that adversely interfere with the activity of the endocrine system (55). Among the numerous ones contaminating our everyday environment, some have been proved to interfere with bone remodeling leading to osteoporotic lesions including phthalates (present in plastics and cosmetics), alkylphenol ethoxylates or APE (added to detergents, additives for fuels and lubricants, perfume fragrances, chemical oils, and flame retardants), perfluoroalkyls (PFAs) (used in the industry of cookware, clothes, carpets, electronics, mechanics), bisphenol A or BPA (present in plastics, food containers, and materials for dental medicine), diethylstilbestrol (DES) (a synthetic estrogen considered in the treatment of selected prostate cancer cases), organotin compounds (used as industrial antifungal agents in textiles, agricultural fungicides, wood preservatives, and antibiofouling agents), and dioxin/dioxin-like compounds (primarily detected in pesticides, waste incineration, and different processes of combustion and paper fabrication) having high affinity for fat stores in animals and humans (9).

Among the few EDs studied for immunomodulatory activity, di(2-ethylhexyl)phthalate or DEHP and its metabolite mono(2-ethylhexyl)phthalate or MEHP act in the BM to inhibit proliferation and induce apoptosis of developing B lymphocytes while suppressing osteogenic MSC commitment in favor of adipogenesis (56, 57). Because increase in BM adipocytes elicits osteoclastogenesis through release of RANKL (58) and inhibits osteoblastogenesis via saturated fatty acids (59), a reduced number of B-cell precursors might contribute to an enhanced osteoclastogenesis reducing the available BM OPG (51, 53). A similar mechanism of bone loss is expected also with the organotin compound, tributyltin, which variably compromises the morphological and functional aspects of all BM niches, and suppresses the proliferation of hematopoietic cells leading to reduced progression of B lymphocytes from the early pro-B to the pre-B stage (60).

Differently, the phthalate ester benzyl butyl phthalate downregulates expression of the histone deacetylases, sirtuins 1 and 3 (61), able to epigenetically inhibit subsets of T lymphocyte (T_H_1 and T_H_17), activate others (Treg), and ensure B lymphocytes survival (62). Thus, it may lead to cancellous bone resorption by a combined hyperactivation of T inflammatory cells and reduced B-cell OPG (7, 12, 35, 36, 40, 46, 51).

In addition, synthetic xenoestrogens such as APE, BPA, and DES may all variably damage survival, maturation, and activation of all immune BM cells (63) via still unknown immunomodulatory effects potentially relevant to the osteoporotic lesions. Finally, the PFA perfluorooctane sulfonic acid exhibits immunotoxicity and impairs MSC commitment (64, 65), supposedly leading to bone loss by reduced control of the BM immune cells on bone anabolism. A similar mechanism is possibly at work also with 2,3,7,8-tetrachlorodibenzo-*p*-dioxin, which blocks the ability of HSC and progenitors to complete a normal differentiation cycle (66). Table 1 summarizes known effects of these and other EDs on cells residing in different bone compartments [from (9, 56, 57, 60, 62, 64–66)], whereas Table 2 provides a tentative integrated view of primary sites of early bone involvement during an inflammatory and/or ED-dependent bone insult based on location and osteomodulatory effects of BM immune cells.

**Table 1:** Effects of EDs on cells in bone compartments.

	**OC**	**OB**	**HSC**	**MSC**	**BMC**	**BL**
PFAs	Abnormal stimulation decreased viability	Increased differentiation decreased viability				
PFOS			Immunotoxicity, reduced differentiation	Immunotoxicity, reduced commitment		
BPA	Increased osteoclastogenesis	Suppressed function and activity		Metabolic alterations, enhanced proliferation, decreased reneval capacity, augmented adipogenic differentiation, alteration of the transcriptomic profile		
APE	Inhibited formation and differentiation	Reduced synthesis of osteocalcin and ALP				
DEHP		Decreased ALP		Decreased Runx2 expression		
MEHP				Suppressed osteogenic commitment, increased lipid accumulation		Inhibited proliferation and induced apoptosis
BBP		Mutagenesis	Reduced haematopoiesis		Reduced cellularity	
DBP		Mutagenesis				
DES	Decreased number and activity					
TBT		Suppressed expression of ALP and osteocalcin, inhibited calcium signaling, and deposition	Suppressed proliferation	Decreased osteogenic capacity, augmented adipogenic differentiation		Reduced progression from pro-B to pre-B
TPhT					Suppressed osteogenic lineage, increased proadipogenic markers	
TCDD	Reduced osteoclastogenesis	Suppressed maturation, reduced ALP and osteocalcin synthesis, reduced osteoblastogenesis	Decreased ability to complete normal differentiation, reduced BM retention and chemotaxis	Reduced Runx2 expression		
PCB	Reduced osteoclastogenesis	Reduced osteoblastogenesis	Reduced BM retention and chemotaxis			
BαP	Decreased activity	Abnormal proliferation				

*B Ly, B lymphocytes; BMC, bone marrow cells; HSC, hematopoietic stem cells; MSC, mesenchymal stromal cells; OB, osteoblasts; OC, osteoclasts; ALP, alkaline phosphatase; APEs, alkylphenol ethoxylates; BαP, benzo[α]pyrene; BBP, benzyl-butyl-phthalate; BM, bone marrow; BPA, 4,4′-isopropylidenediphenol; DBP, di-n-butyl phthalate; DEHP, di(2-ethylhexyl)phthalate; DES, diethylstilbestrol; MEPH, mono(2-ethylhexyl)phthalate; PCB, polychlorinated biphenyls; PFAs, perfluoroalkyls; PFOS, perfluorooctane sulfonic acid; RunX2, Runt-related transcription factor 2; TBT, tributyltin; TCDD, 2,3,7,8-tetrachlorodibenzo-p-dioxin; TPhT, triphenyltin*.

**Table 2:** Microtopography of immune cells in the different bone compartments in basal state and in relation to either an inflammatory or an EDs insult.

**Immune cells**	**Bone sites of residence**	**Bone sites of relocation following inflammation**	**Putative sites of early bone loss by inflammation**	**EDs effect on immune cells**	**Putative sites of early bone damage by EDs immunomodulation**
Monocytes/macrophages	Reticular niche of central and perisinusoidal BM regions; periosteum	Endosteal and subendosteal BM regions	Subendosteal trabecular bone; internal free surface of cortical bone; periosteal bone	[APE, BPA, DES, PFOS, TCDD] –	Full thickness of trabecular bone; subperiosteal cortical bone
Mast cells	Metaphyseal perisinusoidal BM; endosteum of epiphyseal and diaphyseal case	Metaphyseal endosteal BM; endosteum of epiphyseal and diaphyseal case	Subendosteal metaphyseal bone; internal free edge of epiphyseal and diaphyseal compact bone	?	?
T lymphocytes	Follicle-like structures of central and perisinusoidal BM regions	Endosteal and subendosteal BM regions	Subendosteal *trabecular bone; internal free surface of cortical bone*; periosteal bone ?	BBP++ [APE, BPA, DES, PFOS, TCDD] –	See * full thickness of trabecular bone
B lymphocytes and plasma cells	Reticular niche of central and perisinusoidal BM regions	Reticular niche of central and perisinusoidal BM regions	Full thickness of trabecular bone (coupled to compensatory trabecular deposition)	[DHEP, MEHP] –; TBT –; BBP –; [APE, BPA, DES, PFOS, TCDD] –	full thickness of trabecular bone (without compensatory trabecular deposition)
Dendritic cells	All BM regions	all BM regions	In dependance on prevailing effects of other immune cells	[APE, BPA, DES, PFOS, TCDD] –	?

*APE, alkylphenols ethoxylates; BPA, bisphenol A; BBP, benzyl butyl phthalate; DES, diethylstilbestrol; DHEP, di(2-ethylhexyl)phthalate; MEHP, mono(2-ethylhexyl)phthalate; PFOS, perfluorooctane sulfonic acid; TCDD, 2,3,7,8-tetrachlorodibenzo-p-dioxin; ++, immunostimulatory action; –, immunoinhibitory action; ?, unknown. Squared brackets collect EDs with the same immunomodulatory action*.

## Conclusions

A peculiar *in vivo* feature of the bone immune cells is their quite selective segregation in specific BM regions and areas of the cortical bone, both in steady state and upon an inflammatory or ED-dependent insult. In physiological conditions, this cellular distribution is aimed at ensuring functional niches for hematopoiesis and myelopoiesis (23, 50). However, immune cells also regulate bone effector cells (OCs, OBs, MSC), leading to osteocatabolic and osteoanabolic responses selectively bonded to cancellous and/or compact bone. Knowledge of these patterns of response allows for recognition of presumable sites of early bone lesion in the course of an osteoporotic process, and we have here provided a tentative reference sketch integrating the microtopography of immune cells with their osteoinductive and osteolytic effects in basal state and after osteoporotic challenges. Indeed, both primary generalized forms of osteoporosis (postmenopausal and senile) and a number of secondary osteoporotic forms have in common a state of local bone inflammation leading to bone resorption (7, 8). In contrast, EDs may induce either increased bone resorption or inhibition of bone deposition. Collectively, we introduce a space-dependent innovative view of bone remodeling by immune cells in line with the most recent perspectives on the complex spatial logic underling BM function (67). We believe this approach may help to understand how different osteoporotic lesions develop, thus prompting the design of experimental tools for *in vitro* modeling of early phases of the osteoporotic process and related innovative treatments (68).

## Author's Note

RT is under the tenure of the DIMEC - CMG Collaboration Agreement signed on June 27, 2018. Part of these studies have been presented at the National Continuing Medical Education (CME) Program “*Osteoporosi: novità e prospettive*” held at CMG on January 18, 2020. SM and CAB are in training pre-doctoral fellows from the Course of Medical, Veterinary, and Pharmaceutical Biotechnologies at the University of Parma, Parma, Italy.

## Author Contributions

GD, FB, NZ, EC, DD, EQ, and MQ collected and studied the international literature on the topics detailed in the manuscript, and developed clinical studies on jaw osteoporosis. MA, LE, SM, CAB, SP, SS, MS, and AT provided support to analyze the biotechnological relevance of the functional microtopographical pattern ensued from the data acquisition. RT conceived all the ideas and design presented in the text leading the research team involved, wrote, and critically reviewed the entire manuscript. All authors gave the final approval to the manuscript.

## Conflict of Interest

The authors declare that the research was conducted in the absence of any commercial or financial relationships that could be construed as a potential conflict of interest. The reviewer IM declared a past co-authorship with one of the authors to the handling editor.
